# Intrahepatic cholangiocarcinoma arising 28 years after excision of a type IV-A congenital choledochal cyst: report of a case

**DOI:** 10.1007/s00595-012-0387-2

**Published:** 2012-10-23

**Authors:** Takafumi Kumamoto, Kuniya Tanaka, Kazuhisa Takeda, Kazunori Nojiri, Ryutaro Mori, Kouichi Taniguchi, Ryusei Matsuyama, Michio Ueda, Mitsutaka Sugita, Yasushi Ichikawa, Youji Nagashima, Itaru Endo

**Affiliations:** 1 Department of Gastroenterological Surgery, Yokohama City University Graduate School of Medicine, Yokohama, Japan; 2 Department of Molecular Pathology, Yokohama City University Graduate School of Medicine, 3–9 Fukuura Kanazawa-ku, Yokohama, 236-0004 Japan

**Keywords:** Choledochal cyst, Hepatectomy, Intrahepatic cholangiocarcinoma

## Abstract

This report presents a rare case of intrahepatic cholangiocarcinoma (IHCC) arising 28 years after excision of a type IV-A congenital choledochal cyst. The patient underwent excision of a congenital choledochal cyst (Todani’s type IV-A) at 12 years of age, with Roux-en-Y hepaticojejunostomy reconstruction. She received a pancreaticoduodenectomy (PD) using the modified Child method for an infection of a residual congenital choledochal cyst in the pancreatic head at the age of 18. She was referred to this department with a liver tumor 22 years later. Left hemihepatectomy with left-side caudate lobectomy was performed and the tumor was pathologically diagnosed to be IHCC. The cause of the current carcinogenesis was presumed to be reflux of pancreatic juice into the residual intrahepatic bile duct during surgery. This case suggests that a careful long-term follow-up is important for patients with congenital choledochal cysts, even if a separation-operation was performed at a young age, and especially after PD.

## Introduction

Intrahepatic cholangiocarcinoma (IHCC) is the second most common primary liver cancer, and the incidence is increasing [1]. There is a significant association between the presence of congenital choledochal cysts and the development of hepatobiliary malignancies, including IHCC. [2–7] The cause of carcinogenesis in these cases is presumed to be the reflux of pancreatic juice into the bile duct and the accumulation of mixed bile in the biliary system caused by an anomalous junction of the pancreaticobiliary duct.

The recommended standard surgical treatment is the excision of the dilated extrahepatic bile duct, with a hepaticoenterostomy to stop the reflux of pancreatic juice. This is called a “separation-operation.” However, some patients develop biliary cancer long after a separation-operation. The development of intrahepatic cholangiocarcinoma after pancreaticoduodenectomy has not previously been reported in patients with congenital biliary dilation, however, this report presents a case of intrahepatic cholangiocarcinoma arising 28 years after the initial separation-operation for a Todani’s type IV-A congenital choledochal cyst, and 22 years after pancreaticoduodenectomy for infection of a residual congenital choledochal cyst in the pancreatic head.

## Case report

**Fig. 1:**
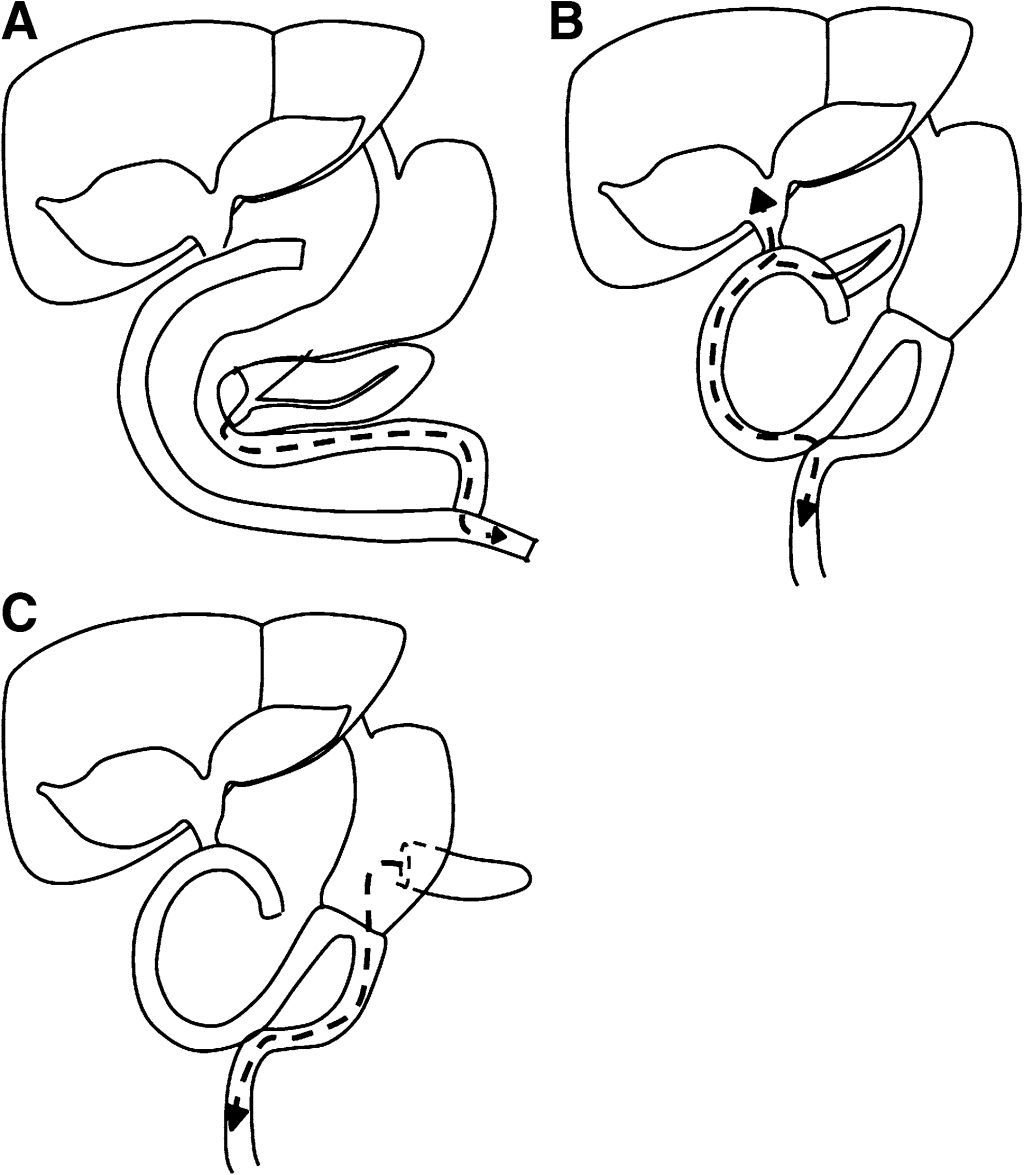
Schematic illustration of the reconstruction. **a** Excision of the extrahepatic dilated bile duct with Roux-en-Y hepaticojejunostomy reconstruction separated the bile from the pancreatic juice flow. *Arrow* with the *broken line* shows the flow of the pancreatic juice. **b** Pancreaticoduodenectomy (PD) using a modified Child method was performed for infection of residual congenital choledochal cyst in pancreatic head. *Arrow* with the *broken line* shows the flow of pancreatic juice that could enter the intrahepatic bile duct. **c** Pancreaticogastrostomy after PD. *Arrow* with the *broken line* shows the flow of pancreatic juice, which is less likely to move backward to the bile duct

A 40-year-old female was referred to this department for further examination of a liver tumor. She had undergone excision of a congenital choledochal cyst (Todani’s type IV-A) at 12 years of age, with Roux-en-Y hepaticojejunostomy reconstruction (Fig. 1a). She underwent a pancreaticoduodenectomy (PD) at the age of 18, using the modified Child method to treat an infection of a residual congenital choledochal cyst in the pancreatic head (Fig. 1b). The patient had been well with no symptoms since her last operation, so she had not undergone regular follow-up during the 18 years since her second operation.

**Fig. 2:**
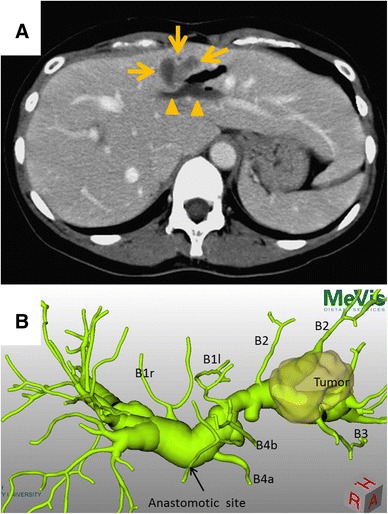
Enhanced abdominal computed tomography and three dimensional-drip infusion cholangiography-computed tomography. **a** *Arrows* show a low-density tumor, 32 mm in diameter, in the lateral section of the liver. *Arrow heads* shows a dilated intrahepatic bile duct. **b** 3D-DIC-CT revealed dilation of the right and left hepatic ducts to 20 mm, and tumor invasion of the B2 + 3 bile duct but not the hepaticojejunostomy

She was diagnosed with a chronic hepatitis C virus (HCV) infection during a medical checkup at the age of 37, and was referred to our hospital for treatment. She had received interferon and ribavirin therapy and finally obtained a sustained viral response, after which she underwent regular follow-up. Follow-up-dynamic abdominal computed tomography (CT) revealed a low-density tumor o the left medial section (Fig. 2a) at the age of 40. Serum carcinoembryonic antigen (CEA) and DUPAN-2 levels were elevated to 6.3 ng/ml and 360 U/ml, respectively. Positron emission tomography-CT (PET-CT) revealed the maximum standardized uptake value (SUVmax) of the liver tumor to be 9.3, with no signs of lymph node metastasis, intraperitoneal dissemination, or hepatic metastasis. 3D-drip infusion cholangiography-CT (3D-DIC-CT) revealed dilation of the right and left hepatic ducts of up to 20 mm and tumor invasion of the B2 + 3 bile duct; the hepaticojejunostomy was not affected by the tumor (Fig. 2b). The tumor was diagnosed to be IHCC.

She underwent a left hemihepatectomy with left-sided caudate lobectomy, preserving the hepaticojejunostomy that had been established in the previous operation. Intraoperative frozen sections of the cut end of the left hepatic duct and lymph node of the hilar lesion revealed no metastasis. Amylase levels in the bile juice of the left hepatic duct were 7621 U/L, and bile juice culture detected the presence of *Enterococcus faecalis*.

**Fig. 3:**
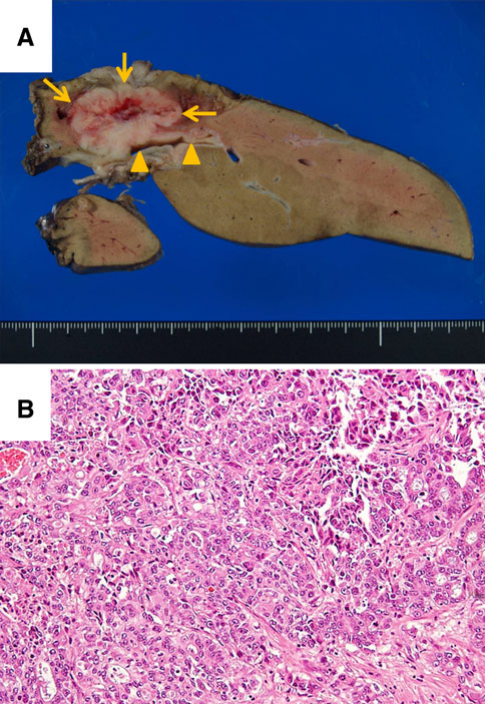
Gross findings of the excised specimen and histopathological findings. **a** *Arrow *shows the tumor and the *arrow head* shows the dilated intrahepatic bile duct. The cut surface of the tumor was 32 mm in diameter, hard and whitish; the margin was somewhat lobulated and the tumor had invaded the B2 + 3 bile duct. **b** The tumor was composed of solid cell nests with occasional glandular spaces. Tumor nuclei were vesicular with coarse chromatin, small nuclei and eosinophilic cytoplasm

The cut surface of the tumor was 32 mm in diameter, hard and whitish; the margin was somewhat lobulated and the tumor had invaded the bile duct of the lateral segment (Fig. 3a). The tumor contained atypical cells with nuclei with enriched chromatin and showed morphological variety. The atypical cells had a poorly glandular arrangement (Fig. 3b).

The patient received postoperative adjuvant systemic chemotherapy with gemcitabine. There was no recurrence 10 months after surgery.

## Discussion

The development of biliary cancer is a major complication in patients with congenital choledochal cysts, with an incidence of hepatobiliary malignancies associated with congenital choledochal cysts ranging from 2.5 to 28 % [2–7]. Although the mechanism of carcinogenesis has not been fully elucidated, it is reported that the carcinogenetic process is caused by repeated damage and restoration of the biliary epithelium by a mutual countercurrent of pancreatic and bile juice. The regenerated epithelium gradually produces a variant accompanied by cellular atypical changes, as well as mutations of the K-ras and p53 genes [8]. These processes may lead to mucosal metaplasia and biliary tract malignancy.

Excision of the entire extrahepatic bile duct and hepaticoenterostomy are recommended to prevent the development of biliary carcinoma, because it separates bile from pancreatic juice flow. However, some patients develop intrahepatic cholangiocarcinoma long after the separation-operation. Kobayashi et al. [8]. reported biliary tract cancer before and after separation-operations for patients with congenital biliary dilation, and concluded that the relative risk in the post-surgery group was still higher than in the general population, although it was decreased by approximately 50 % after the separation-operation. This suggests that the epithelium of the remnant bile duct wall may have already progressed to a precancerous stage by the time of surgery, and that genetic changes may have taken place or continued during the postoperative period. Furthermore, all patients in this previous study that developed bile duct carcinoma after surgery had a Todani’s type IV-A dilation, characterized by narrowing of the peripheral bile duct and a dilated pathologic bile duct. [9] A complete resection of a dilated pathologic intrahepatic bile duct is not a straightforward procedure, and thus the risk of developing cancer remains high. This was also true in the current case, in which the patient was diagnosed with Todani’s type IV-A dilation and the residual dilated intrahepatic duct was detected by 3D-DIC-CT.

Patients who undergo biliary-enteric anastomosis are thought to be at risk for developing IHCC after surgery for benign disease, as the reflux of activated pancreatic juice and bacterial contamination can cause chronic inflammation and carcinogenic processes. Tocchi et al. [10] reported that the incidence of cholangiocarcinomas after choledochoduodenostomy or hepaticojejunostomy for benign disease is 7.6  and 1.9 %, respectively, and this significant difference occurs because the activated pancreatic juice can more easily flow back to the biliary tract in a choledochoduodenostomy. Therefore, the reflux of activated pancreatic juice might be the strongest carcinogenic factor.

Re-exposure to pancreatic juice may have been one of the causes of cancer in the current case. The residual dilated intrahepatic bile duct appeared to have been stimulated by a mutual countercurrent of pancreatic and bile juice and by intestinal bacteria, because *E. faecalis* was detected in a culture of the bile juice and amylase levels were 7621 U/L in the bile juice of the left hepatic duct. Therefore, pancreaticogastrostomy is recommended for patients with congenital choledochal cysts after PD, because it is more difficult for pancreatic juice to flow backward to the bile duct during this procedure (Fig. 1 c).

Re-anastomosis of the hepaticojejunostomy using another Roux-en-Y to prevent pancreatic juice from flowing backward to residual dilated right hepatic duct during resection of the IHCC was planned in the current case, because the patient was relatively young. However, the adhesion of the hepatic hilum and jejunum was strong and there was a risk of damage to the right hepatic artery. Therefore, only a left hemihepatectomy with left-sided caudate lobectomy was performed, preserving the hepaticojejunostomy that had been established in the previous operation.

Infection with hepatitis B virus or HCV is suggested to be involved in the pathogenesis of IHCC. A large cohort study revealed that HCV infection conferred a more than two fold elevated risk of developing IHCC, [11] while Yamamoto et al. [12] reported that nodular IHCC appears to be related to hepatitis viral infection and could be detected at an early stage by following up cases of chronic hepatitis and cirrhosis. The current patient had not undergone regular follow-up for 18 years after her second operation. However, she underwent periodic medical check-ups after being diagnosed with chronic HCV infection that allowed IHCC to be detected early and a potentially curative operation to be performed.

An extensive literature search revealed ten reports describing IHCC arising after surgery for a congenital choledochal cyst (Table 1). The 11 patients, including the current case, included five males, five females and one case of unknown gender, ranging in age from 16 to 66 years. The mean period between the primary operation and the development of cancer was 15.3 years (2–34 years) and the type of dilation was Todani’s type IV-A in eight patients and type I in one patient. The IHCC was resected successfully in only four of these patients, three had an unresectable advanced tumor and one had a resectable tumor that was inoperable due to poor liver function. A periodic medical check-up is important for detecting the tumor at an early stage; since cholangiocarcinoma is not characterized by distinct clinical symptoms until its advanced late stages.

**Table 1:** Published cases of intrahepatic cholangiocarcinoma arising several years after surgery for a congenital choledochal cyst

Case	Sex	Age (years)	Todani tumor type	Years after surgery	Site of carcinoma	Resectability	First author
1	F	58	IV-A	7	Lt	Unresected	Gallagher [13]
2	F	38	IV-A	17	–	Unresected	Chandhuri [14]
3	M	33		20	Rt	Unresected	Cohen [15]
4	–	–	IV-A	2	Rt	Resected	Scudamore [16]
5	M	29	IV-A	3	Bil	Unresected	Joseph [17]
6	F	18	IV-A	2.4	Lt	Unresected	Kobayashi [9]
7	F	52	I	10	A	Resected	Goto [18]
8	M	46	IV-A	26	M	Unresected	Suzuki [19]
9	M	44	IV-A	34	Lt	Resected	Shimamura [20]
10	M	66	–	20	Rt	Unresected	Matsuura [21]
11	F	40	IV-A	28	M	Resected	Present case

This report presented a case of intrahepatic cholangiocarcinoma arising 28 years after the initial operation of excision of a Todani’s type IV-A congenital choledochal cyst with reconstruction by Roux-en-Y hepaticojejunostomy. The patient had multiple possible risk factors for developing IHCC, including remaining IV-A congenital biliary dilation, subsequent modified Child PD that induced re-exposure of pancreatic juice, and chronic HCV infection. Careful long-term follow-up is therefore recommended for high risk patients even after separation-operation.

## References

[1] Yamamoto M, Ariizumi S (2011). Surgical outcomes of intrahepatic cholangiocarcinoma. Surg Today.

[2] Nagorney DM, McIlrath DC, Adson MA (1984). Choledochal cysts in adults: clinical management. Surgery.

[3] Kagawa Y, Kashihara S, Kuramoto S, Maetani S (1978). Carcinoma arising in a congenitally dilated biliary tract: report of a case and review of the literature. Gastroenterology.

[4] Todani T, Tabuchi K, Watanabe Y, Kobayashi Y, Kobayashi T (1979). Carcinoma arising in the wall of congenital bile duct cysts. Cancer.

[5] Yamaguchi M (1980). Congenital choledochal cysts: analysis of 1,433 patients in the Japanese literature. Am J Surg.

[6] Deaiel DJ, Rossi RL, Munson JL, Braasch JW, Silverman ML (1986). Management of bile duct cysts in adults. Arch Surg.

[7] Todani T, Watanabe Y, Urushihara N, Morotomi Y, Maeba T (1994). Choledochal cyst, pancreatobiliary malunion, and cancer. J Hepatobiliary Pancreat Surg.

[8] Kasuya K, Nagawaka Y, Matsudo T, Ozawa T, Tsuchida A (2009). p53 gene mutation and p53 protein overexpression in a patient with simultaneous double cancer of the gallbladder and bile duct associated with pancreaticobiliary maljunction. J Hepatobiliary Pancreat Surg.

[9] Kobayashi S, Asano T, Yamasaki M, Kenmochi T, Nakagohri T (1999). Risk of bile duct carcinogenesis after excision of extrahepatic bile ducts in pancreaticobiliary maljunction. Surgery.

[10] Tocchi A, Mazzoni G, Liotta G, Lepre L, Gassini D (2001). Late development of bile duct cancer in patients who had biliary-enteric drainage for benign disease: a follow-up study of more than 1,000 patients. Ann Surg.

[11] El-Serag HB, Engels EA, Landgern O, Chiao E, Henderson L, Amaratunge HC (2009). Risk of hepatobiliary and pancreatic cancers after hepatitis C virus infection: a population-based study of U.S. veterans. Hepatology.

[12] Yamamoto M, Takasaki K, Nakano M, Saito A (1998). A minute nodular intrahepatic cholangiocarcinoma. Cancer.

[13] Gallagher PJ, Millis RR, Mitchinson MJ (1972). Congenital dilatation of the intrahepatic bile ducts with cholangiocarcinoma. J Clin Pathol.

[14] Chaudhuri PK, Chaudhuri B, Schuler JJ, Nyhus LM (1982). Carcinoma associated with congenital cystic dilation of bile ducts. Arch Surg.

[15] Cohen GP, O’Donnell C (1992). Malignant change within surgically drained choledochal cysts. Australas Radiol.

[16] Scudamore CH, Hemming AW, Teare JP, Fache JS, Erb SR (1994). Surgical management of choledochal cysts. Am J Surg.

[17] Joseph VT (1990). Surgical techniques and long-term results in the treatment of choledochal cyst. J Pediatr Surg.

[18] Goto N, Yasuda I, Uematsu T, Kanemura N, Takai S (2001). Intrahepatic cholangiocarcinoma arising 10 years after the excision of congenital extrahepatic biliary dilation. J Gastroenterol.

[19] Suzuki S, Amano K, Harada N, Tanaka S, Hayashi T, Suzuki M (1988). A case of intrahepatic cholangiocarcinoma arising 26 years after excision of congenital biliary dilation (in Japanese with English abstract). Jpn J Gastroenterol Surg.

[20] Shimamura K, Kurosaki I, Sato D, Takana K, Yokoyama N, Sato Y (2009). Intrahepatic cholangiocarcinoma arising 34 years after excision of a type IV-A congenital choledochal cyst: report of a case. Surg Today.

[21] Matsuura H, Inui K, Wakabayashi T, Okushima K, Miyoshi H, Nakamura Y (2009). A cholangiocarcinoma detected while treating intrahepatic stones 20 years after operative biliary diversion for congenital dilatation of the bile duct. JJBA.

